# Supplementary material for: Different genetic mechanisms mediate spontaneous versus UVR-induced malignant melanoma
Source: eLife. 2019 Jan 25;8:e42424. doi: 10.7554/eLife.42424 (PMC6428585; doi:10.7554/eLife.42424)
Supplement: Supplementary file 2. — Unprocessed raw counts based on Fragments per kilo base per million mapped reads. Table shows results for whole skin from 8 weeks old mice of the following strains; AJ, NOD, 129S, FVB, and C57BL/6 [file elife-42424-supp2.pptx]

## Slide 1
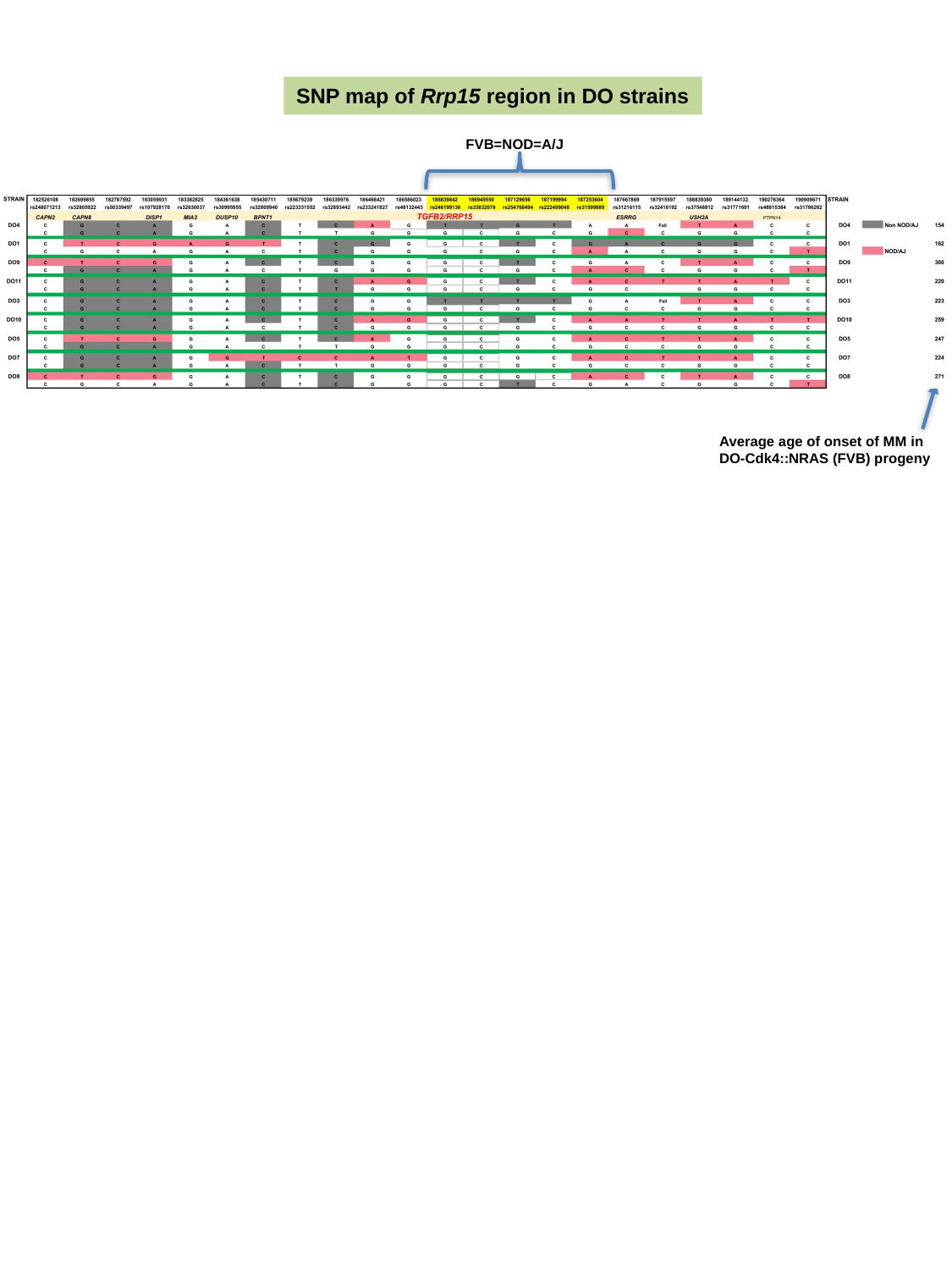

SNP map of Rrp15 region in DO strains
FVB=NOD=A/J
Average age of onset of MM in
DO-Cdk4::NRAS (FVB) progeny
